# Linking gut microbiome to HIV-1 reservoir size in people living with HIV

**DOI:** 10.1186/s13099-026-00828-2

**Published:** 2026-03-30

**Authors:** Oscar Kieri, Aswathy Narayanan, Bianca B Jütte, Peter Svensson, Soo Aleman, Anders Sönnerborg, Shilpa Ray, Piotr Nowak

**Affiliations:** 1 Department of Infectious Diseases, Karolinska University Hospital Huddinge, Stockholm, Sweden; 2 Department of Medicine Huddinge, Center for Infectious Medicine, Clinical Infectious Diseases, Karolinska Institutet, Stockholm, Sweden; 3 Department of Medicine Huddinge, Center for Infectious Medicine, Karolinska Institutet, Stockholm, Sweden; 4 Department of Translational Pharmacology, Medical School OWL, Bielefeld University, Bielefeld, Germany; 5 Department of Clinical Microbiology, Karolinska University Hospital, Stockholm, Sweden

**Keywords:** HIV-1 Reservoir, Gut microbiome, Intact Proviral DNA Assay, Shotgun metagenomic sequencing, Microbial metabolic pathways, Alpha diversity

## Abstract

The gut microbiome is altered during HIV-1 infection and contributes to immune dysfunction and inflammation in people living with HIV (PLWH), these changes may persist despite effective antiretroviral therapy (ART). We explored the associations between the fecal gut microbiome and blood HIV-1 reservoir size in PLWH (*n* = 30) on long-term ART. The intact proviral DNA assay (IPDA) and shotgun metagenomic sequencing were performed to identify microbial species and metabolic pathways associated with the size of the HIV-1 reservoir. PLWH with a smaller intact reservoir exhibited lower evenness compared to individuals with a larger intact reservoir. We found that *Phocaeicola plebeius* and *Lachnospira sp000437735* were significantly enriched in individuals with a smaller intact reservoir and lower intact-to-total proviral ratio, respectively. We observed a negative association between *Faecalibacterium prausnitzii* and a positive association of *Prevotella copri*, with the intact proviral reservoir size. Additionally, the metabolic pathways of glycolysis and branched-chain amino acid biosynthesis were enriched in individuals with larger reservoir. HIV reservoir size in blood is associated with gut microbiome evenness, specific metabolic pathways and microbial signatures, including *Lachnospira*,* Prevotella*, and *Faecalibacterium*. Our findings underscore the potential role of the gut microbiome in viral persistence, raising the possibility that modulating microbial composition could influence the HIV reservoir.

**Supplementary Information:**

The online version contains supplementary material available at 10.1186/s13099-026-00828-2.

## Introduction

The gut microbiome plays a critical role in modulating immune responses and systemic inflammation in people living with HIV (PLWH), and these two key factors influence the persistence of the HIV-1 reservoir [1]. During HIV-1 infection, the gut-associated lymphoid tissue (GALT) becomes a major site for viral replication and immune activation [2, 3]. This process compromises gut barrier integrity, leading to microbial translocation and sustained systemic inflammation [4].

Although antiretroviral therapy (ART) effectively suppresses viral replication and has dramatically reduced HIV-related morbidity and mortality, it does not fully restore gut immune homeostasis or repair the structural and functional damage within the GALT [5, 6]. Additionally, the gut microbiome is altered during HIV infection, and studies in individuals receiving long-term ART have reported variable findings, with some reporting persistent features of dysbiosis and others suggesting restoration in association with immune reconstitution [7–12]. Lastly, ART alone is insufficient to eliminate the latent HIV reservoir, which persists primarily in lymphoid tissues and represents the major obstacle to achieving a definitive cure [13, 14].

Persistent gut barrier dysfunction allows microbial-derived products such as lipopolysaccharides (LPS) and bacterial DNA to enter systemic circulation, driving chronic immune activation [4, 15]. This persistent immune stimulation promotes CD4^+^ T cell activation, facilitating maintenance and potential expansion of the HIV reservoir, which is predominantly harbored within the GALT [16]. Additionally, microbial metabolites could in turn affect the kynurenine pathway, whose enzymatic activity has been associated with the size of the HIV reservoir [17]. Despite these insights, the direct influence of gut microbiome composition and impact on the size and dynamics of the HIV reservoir remains incompletely understood, and robust clinical data are lacking.

Our previous research has shown distinct differences in gut microbiome composition and functional capacity among viremic individuals, ART-treated individuals, and elite controllers [18–20]. These individuals also differ in reservoir size and suggest that the microbiome may shape the HIV reservoir [21, 22]. To explore the potential role of the gut microbiome in HIV persistence, the primary objective of our current study was to investigate the interplay between the gut microbiome and the HIV-1 reservoir. Specifically, we aimed to identify microbial taxa and functional pathways that correlate with the size of the intact HIV-1 reservoir, with the goal of uncovering novel microbiome-based targets for reservoir modulation.

## Materials and methods

### Study cohort

Individuals included in this study were part of the COVAXID trial, an open-label, non-randomized prospective clinical trial at the Karolinska University Hospital, Stockholm, Sweden, to investigate the safety and clinical efficacy of the mRNA BNT162b2 vaccine (Comirnaty^®^, Pfizer/BioNTech) in immunocompromised individuals [23]. The ethical permit was granted by the Swedish Ethical Review Authority (ID 2021 − 00451, ID 2023-05153-02, ID 2025-00036-02), and all participants provided written informed consent. All methods of this study were performed in accordance with the Declaration of Helsinki and The Good Clinical Practice guidelines. The trial was registered at the European Union Drug Regulating Authorities Clinical Trials Database (EudraCT 2021-000175-37) and ClinicalTrials.gov (NCT04780659) by Feb 9, 2021, and Feb 19, 2021, respectively. The trial was also approved by the Swedish Medical Products Agency (ID 5.1-2021-5881). The original trial protocol is available via the SciLifeLab Data Repository.

PLWH, aged 18–85, followed at the outpatient HIV clinic, eligible for COVID-19 vaccination, were screened for inclusion in the trial. Recruitment started on Feb 15, 2021 and follow-up ended Oct 15, 2021. The trial was fully recruited as intended in the study plan. Ninety PLWH on ART were enrolled in the trial, with fecal and blood samples for reservoir analysis collected from 39 participants, prior to vaccination. After excluding nine individuals who had received antibiotic treatment within three months prior to inclusion [24], the analysis was conducted on the remaining 30 participants. Clinical and laboratory characteristics were obtained from electronic health records. Basic dietary information (omnivorous, vegetarian) and body mass index (BMI) were collected at inclusion in the study. Following reservoir analysis, described below, participants were stratified into groups of higher and lower levels of intact proviral HIV-1 DNA and ratio of intact-to-total proviruses, with the median as the divider.

### Reservoir analysis

Blood samples were collected from the participants for reservoir analysis. PBMCs were isolated by density gradient separation, with a median of 6.1 million cells/sample (IQR 4.2, 8.0). Resting CD4^+^ T cells (rCD4^+^ T cells) were isolated from the obtained PBMCs by a two-step magnetic isolation using the Miltenyi Biotec magnetic associated cell sorting (MACS) platform. For the first step, the CD4^+^ negative cell isolation kit (Miltenyi Biotec, Cat# 130-096-533) was used according to the manufacturer’s protocol. Secondly, rCD4^+^ T cells were negatively selected with magnetic beads against the activation markers CD25, CD69 and HLA-DR. DNA was extracted by the QIAamp DNA Mini Kit (Qiagen, Cat#51304), a median of 1.6 µg (IQR 1.0, 1.9) DNA was extracted per sample. No significant differences in the number of cells and amount of extracted DNA were observed between groups. The DNA was used to quantify the HIV-1 reservoir (intact, 5′ (*Ψ)* deleted, and 3′ (*env*) deleted/hypermutated proviruses) using the Intact Proviral DNA Assay (IPDA), which has been previously described [25]. During calculation of intact proviruses, each value was corrected for the DNA shearing in that specific sample.

For IPDA, digital droplet PCR (ddPCR) was performed with the QX200 Droplet Digital qPCR System (Bio-Rad). Each reaction consisted of 20 µL, containing 10 µL Supermix for Probes without dUTP (Bio-Rad, Cat# 1863024), 900 nM primers, 250 nM probe (labelled with HEX or FAM), and 8 µL undiluted cellular DNA. Blood from individuals without HIV (*n* = 4) were used in parallel as negative controls samples. As a positive control we used a J-Lat cell lines 5A8 ^26,27^. Droplets were generated using the QX200 droplet generator. Emulsified PCR reactions were performed with a C1000 Touch thermal cycler (Bio-Rad), with the following protocol: 95 °C for 10 min, followed by 40 cycles of 94 °C for 30 s and 57 °C for 60 s, and a final droplet cure step of 10 min at 98 °C. Each well was then read with a QX200 Droplet Reader (Bio-Rad). Droplets were analyzed with QuantaSoft version 1.5 (Bio-Rad) software in the absolute quantification mode.

### Fecal sample processing

The fecal samples were collected in RNA/DNA shield (Stratec, Germany), and kept at -80 °C until parallel analysis of all samples was performed. Subsequently, DNA was extracted using QIAamp PowerFecal Pro DNA Kits – Stool/Gut DNA Extraction (Qiagen) and sequenced on the Illumina Novaseq6000 platform. DNA extraction was performed 14 months after sample collection. Prior to library preparation and sequencing, the concentration of the samples was quantified using Qubit and confirmed to be above 100 ng, as required by the sequencing method. Library preparation and construction were performed using Illumina DNA (Flex). We obtained approximately 40 million sequencing reads that were generated across all the samples.

### Sequence analysis

The paired-end sequences obtained from the sequencing platform were quality-checked using FastQC [28]. Adapter and low-quality reads (Phred score < 30) were removed using Trim Galore (v0.6.4) [29]. Further, host DNA contamination was removed from the fastq sequences using a combination of Bowtie2 (v2.3.5.1) [30, 31], SAMtools (v1.19) [32], and BEDtools (v2.29.2) [33]. Pre-processed reads were then analyzed for taxonomic classification using MetaPhlAn4 [34], and abundant functional pathways were predicted from the metagenomic data using HUMAnN3 [35]. HUMAnN3 is a bioinformatics pipeline that profiles the functional and metabolic potential of microbial communities directly from shotgun metagenomic sequencing data. A filtering step was included to remove low-prevalence and low-abundance bacteria from the data using the R function *phyloseq_filter_prevalence*, with a prevalence threshold of 10%. The samples sequenced had in mean 48.1 million reads before filtering and in mean 41.1 million reads after filtering. The alpha diversity indices such as Observed, Chao1, Shannon, Simpson and InvSimpson were performed to calculate the richness and evenness of the samples. Linear discriminant effect size (LEfSe) was employed to identify significant microbial compositions between groups. Spearman correlation was performed using the R package psych (v2.4.3) [36] to analyze the association between microbiota at the species level, clinical parameters and HIV-1 reservoir, with p-values adjusted using false discovery rate (FDR) correction. The plots were visualized using the R package ggplot2 (v3.5.1) [37].

## Results

### Study participants

The study included 30 PLWH with a median age of 54 years (IQR 44, 68), 40% of whom were female. All participants were on ART at inclusion, with a median treatment duration of eight years (IQR 4, 15). The majority (83%) were treated with integrase strand transfer inhibitors (INSTI), and 90% had a HIV RNA viral load of less than 50 copies/mL. In viremic participants the median HIV RNA viral load was 62 copies/mL (range 56–308). The median CD4^+^ T cell count was 630 cells/mm³ (IQR 290, 720), with a CD4^+^/CD8^+^ ratio of 1.05 (IQR 0.55, 1.40). Nearly half (47%) had a nadir CD4^+^ T cell count below 200 cells/mm³, with a median nadir CD4^+^ T cell count of 260 cells/mm³ (IQR 80, 410) (Table 1).

**Table 1:** Characteristics according to intact HIV-1 proviral reservoir size at inclusion

Characteristics	High *N* = 15^*1*^	Low *N* = 15^*1*^	Overall *N* = 30^*1*^	*p*-value^2^
Sex				0.14
Male	11 (73%)	7 (47%)	18 (60%)	
Female	4 (27%)	8 (53%)	12 (40%)	
Age (years)	54 (44, 68)	54 (41, 63)	54 (44, 68)	0.8
CD4^+^ T-cell count	320 (240, 720)	642 (570, 770)	630 (290, 720)	0.042
Nadir CD4^+^ T-cell count	140 (58, 280)	370 (220, 470)	261 (80, 410)	0.007
CD4^+^/CD8^+^ ratio	0.58 (0.37, 1.31)	1.29 (0.96, 2.10)	1.05 (0.55, 1.40)	0.036
HIV RNA < 50 copies/ml	13 (87%)	14 (93%)	27 (90%)	0.54
Duration of ART (years)	10 (4, 15)	7 (4, 20)	8 (4, 15)	0.7
ART regimen				> 0.9
INSTI-based	12 (80%)	13 (87%)	25 (83%)	
NNRTI-based	3 (20%)	2 (13%)	5 (17%)	
BMI (kg/m^2^)	24.0 (21.0, 27.0)	25.0 (22.9, 29.0)	25.0 (22.9, 27.0)	0.8
Diet				> 0.9
Omnivorous	14 (93%)	14 (93%)	28 (93%)	
Others/Unknown	1 (6.7%)	0 (0%)	1 (3.3%)	
Vegetarian	0 (0%)	1 (6.7%)	1 (3.3%)	
Ethnicity				> 0.9
Asian	3 (20%)	3 (20%)	6 (20%)	
Black	2 (13%)	2 (13%)	4 (13%)	
Caucasian	10 (67%)	9 (60%)	19 (63%)	
Latino	0 (0%)	1 (6.7%)	1 (3.3%)	
Route of transmission				0.5
Heterosexual	8 (53%)	10 (67%)	18 (60%)	
MSM	7 (47%)	5 (33%)	12 (40%)	
Intact provirus	196 (87, 391)	34 (27, 45)	60 (34, 196)	< 0.001
5´defective provirus	335 (142, 1,069)	211 (125, 347)	281 (142, 832)	0.15
3´defective provirus	318 (202, 902)	120 (91, 169)	190 (104, 342)	< 0.001
Total defective provirus	641 (385, 2,097)	318 (243, 624)	527 (272, 1,099)	0.037
Total provirus	729 (522, 2,542)	363 (272, 677)	632 (307, 1,138)	0.011
Ratio intact-to-total provirus	0.18 (0.15, 0.29)	0.08 (0.04, 0.12)	0.12 (0.08, 0.19)	< 0.001

ART, antiretroviral therapy; INSTI, integrase strand transfer inhibitor; NNRTI, non-nucleoside analogue reverse transcriptase inhibitor; MSM, men who have sex with men; BMI, body mass index. CD4^+^ T-cell count (cells/mm^3^); Nadir CD4^+^ T-cell count (cells/mm^3^); provirus (proviral HIV DNA copies/million resting CD4^+^T-cells)

^1^ n (%); Median (Q1, Q3)

^2^ Pearson’s Chi-squared test; Wilcoxon rank sum test; Fisher’s exact test; Wilcoxon rank sum exact test

### Reservoir analysis

We quantified the intact and defective (5′ deleted and 3′ deleted/hypermutated) proviral HIV-1 reservoir per million rCD4^+^ T cells in blood using IPDA (Supplementary Fig. 1A). The median intact reservoir size was 60 copies/million rCD4^+^ T cells (IQR 34, 198), while the defective reservoir contained 282 (IQR 141, 874) copies of 5′-deleted proviruses and 190 (IQR 103, 350) copies of 3′-deleted/hypermutated proviruses per million rCD4^+^ T cells. The estimated median total reservoir size (intact and defective) was 632 (301–1144) and correlated with the amount of intact provirus (Spearmans’s, *r* = 0.68, p = < 0.0001) (Supplementary Fig. 1D), with a median ratio of intact-to-total provirus of 0.13 (IQR 0.08, 0.2) (Supplementary Fig. 1C). 

In order to elucidate relationships between the HIV-1 reservoir size and the gut microbiome, we stratified PLWH based on higher and lower levels of intact proviral HIV-1 DNA and ratio of intact-to-total proviruses, a potential indicator of reservoir decay, using the median as divider (Supplementary Fig. 1B, 1 C). PLWH with a larger intact reservoir size had a significantly lower CD4^+^ T cell count, with a median of 320 cells/mm^3^ (IQR 240, 720), compared to individuals with a smaller intact reservoir size, who had a median of 642 cells/mm^3^ (IQR 570, 770) (*p* = 0.04). Similar relationships were observed for the nadir CD4^+^ T cell count and the CD4^+^/CD8^+^ ratio. In the groups of higher and lower ratio of intact-to-total provirus a similar trend was observed. Despite the differences in immune status, the duration and type of ART did not differ between the reservoir groups, suggesting that these factors are unlikely to account for the observed differences. Additionally, there were no significant differences in age, sex distribution, BMI, diet, ethnicity, or mode of transmission. Antibiotic use was absent, as individuals who had received antibiotics within three months prior to inclusion were excluded from the study. The detailed characteristics are presented in Table 1.

### Alterations in bacterial diversity and composition in relation to intact proviral reservoir and intact-to-total proviral ratio

**Fig. 1:**
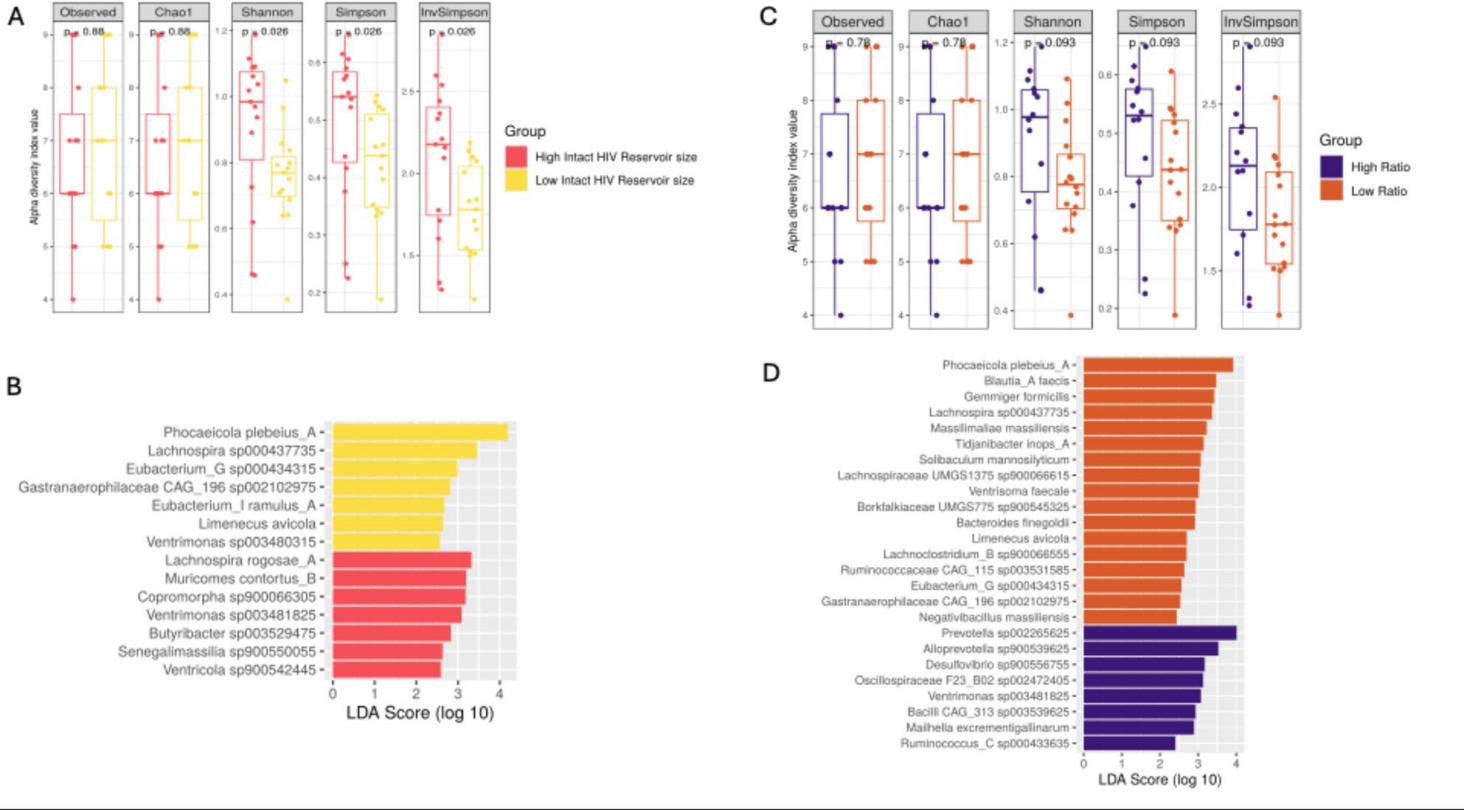
Differences in the bacterial diversity and richness according to intact proviral reservoir size (**A**) and ratio of intact-to-total proviruses (**C**) between groups of high and low. Linear discriminant effect size analysis (LEfSe) at the species level showing the significantly abundant organisms (LDA score ≥2) between high and low groups based on the intact proviral reservoir size (**B**) and ratio of intact-to-total proviruses (**D**)

We observed no significant difference in richness (Observed *p* = 0.88; Chao1 *p* = 0.88) between the groups of higher and lower intact reservoir size (Fig. 1A). However, individuals within the group of higher intact proviral reservoir size showed significantly higher diversity indices of Shannon (*p* = 0.03) and Simpson (*p* = 0.03), suggesting a higher evenness in this group. On the other hand, this was not observed in the intact-to-total proviral ratio groups (Fig. 1C) and in regression analysis we could not demonstrate any linear associations between alpha diversity and intact reservoir size (Supplementary Fig. 2).

We observed a significantly greater abundance of *Lachnospira rogosae A* (*p* = 0.04), *Muricomes contortus B* (*p* = 0.04), and *Copromorpha sp900066305* (*p* = 0.02) in the group with a high intact reservoir. On the contrary, in the group with a low intact reservoir, we found significant enrichment of *Phocaeicola plebeius A* (*p* = 0.04), *Lachnospira sp000437735* (*p* = 0.02) and *Eubacterium G sp000434315* (*p* = 0.02) (Fig. 1B). Furthermore, *Prevotella sp002265625* (*p* = 0.02), *Alloprevotella sp900539625* (*p* = 0.01), and *Desulfovibrio sp900556755* (*p* = 0.04) were significantly abundant in individuals with a high ratio. On the other hand, individuals with a low ratio showed a significant increase in *Phocaeicola plebeius A* (*p* = 0.04), *Lachnospira sp000437735* (*p* = 0.003), *Blautia* A *faecis* (*p* = 0.04), and *Gemmiger formicilis* (*p* = 0.04) (Fig. 1D).

### Association between microbiota and intact proviral reservoir size and intact-to-total proviral ratio

**Fig. 2:**
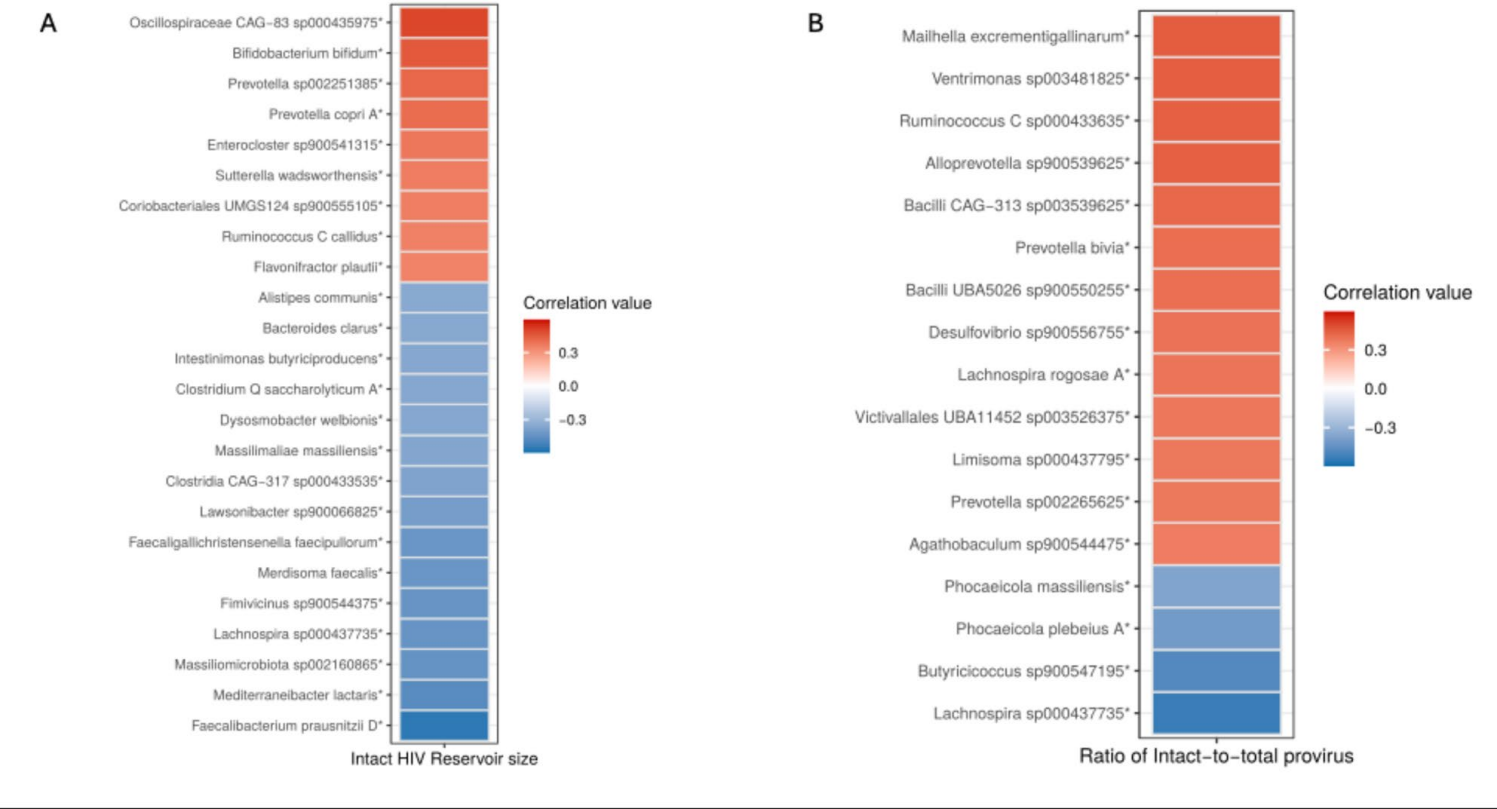
Spearman correlation analysis between microbiota at species level and intact proviral reservoir size (**A**) and ratio of intact-to-total proviruses (**B**)

We found several significant correlations between different bacterial species and the level of intact HIV-1 DNA. Notably, we observed a negative association of *Faecalibacterium prausnitzii D* (*p* = 0.001), *Lachnospira sp000437735* (*p* = 0.01), and *Bacteriodes clarus* (*p* = 0.05) with intact proviral reservoir size. On the contrary, a significant positive association was observed with *Oscillospiraceae CAG-83 sp000435975* (*p* = 0.004), *Bifidobacterium bifidum* (*p* = 0.01), *Prevotella copri A* (*p* = 0.02), and *Sutterella wadsworthensis* (*p* = 0.04) (Fig. 2A). Furthermore, we observed a significant positive association of the ratio of intact-to-total proviruses with *Ventrimonas sp003481825* (*p* = 0.01*)*, *Prevotella bivia* (*p* = 0.02) and *Lachnospira rogosae A* (*p* = 0.03) while taxa such as *Lachnospira sp000437735* (*p* = 0.01), *Phocaeicola plebeius A* (*p* = 0.02), and *Phocaeicola massiliensis* (*p* = 0.04) were negatively associated with the ratio of intact-to-total proviruses (Fig. 2B).

### Relationship of metabolic pathways with HIV-1 reservoir size and intact-to-total proviral ratio

**Fig. 3:**
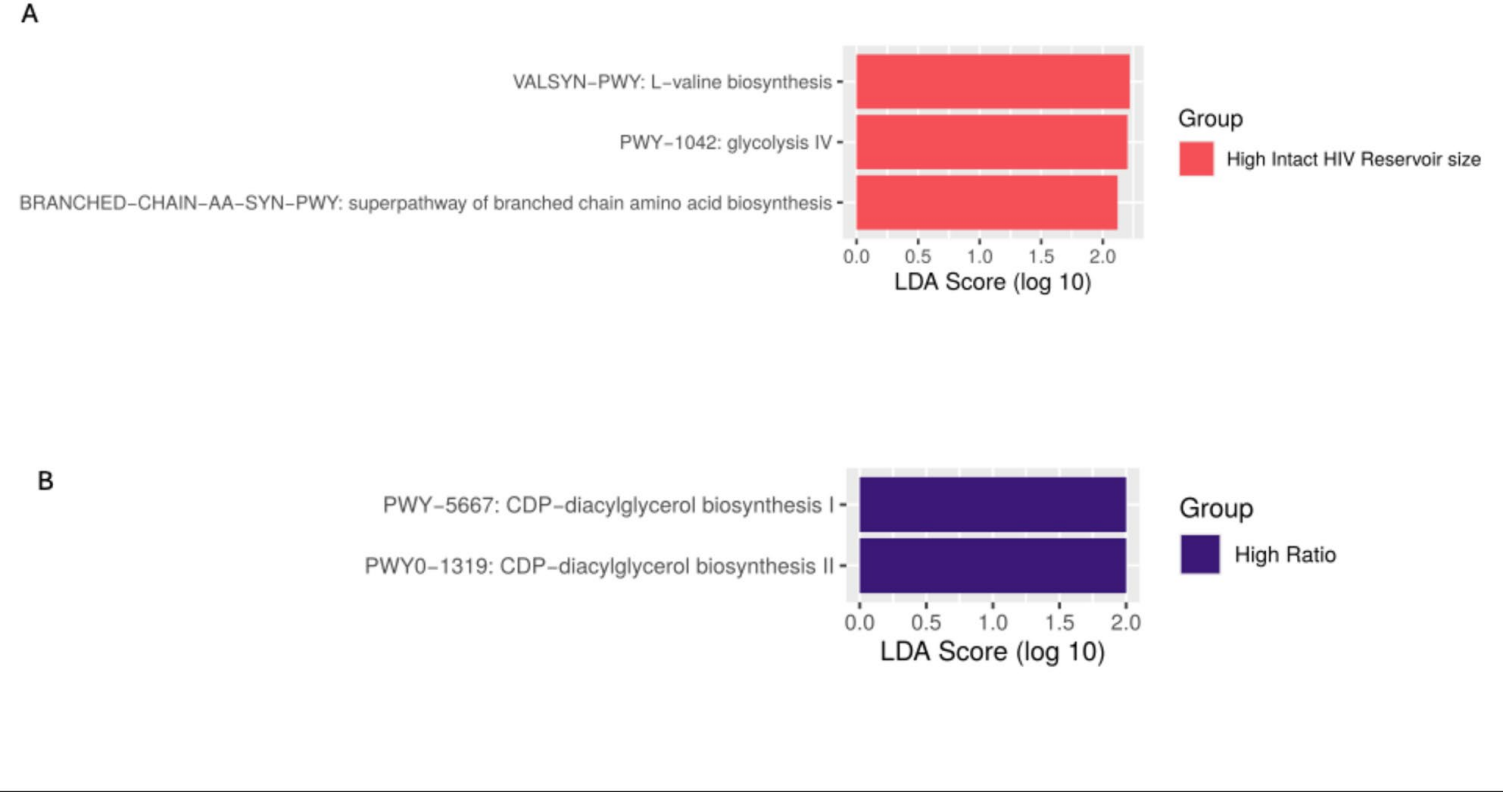
Linear discriminant effect size analysis (LEfSe) showing the significantly abundant pathways (LDA score ≥2) in high and low groups based on intact proviral reservoir size (**A**) and intact-to-total proviruses (**B**)

We further investigated if the functional pathways of the metabolic traits, predicted from the metagenome sequence, were related to the intact proviral HIV-1 reservoir size and intact-to-total proviral ratio. Our analysis revealed a significantly higher abundance of L-valine biosynthesis (*p* = 0.02), glycolysis IV (*p* = 0.04) and super-pathway of branched-chain amino acid biosynthesis (*p* = 0.04) in PLWH within the group with a high intact proviral reservoir (Fig. 3A). Additionally, we observed a significant abundance of CDP-diacylglycerol biosynthesis I and II (*p* = 0.04) in PLWH within the high ratio group (Fig. 3B).

### Bacteroidales/Clostridiales ratio in PLWH with respect to intact proviral reservoir and intact-to-total proviral ratio

As a previous study found an inverse correlation between the *Bacteroidales/Clostridiales* ratio and total HIV-1 reservoir size [38], we explored whether similar patterns were present in our cohort. However, we did not observe significant differences in *Bacteroidales*,* Clostridiales*, and *Bacteroidales/Clostridiales* ratio between the groups of higher and lower intact reservoir size and ratio of intact-to-total proviruses in our study cohort (Fig. S3 A, B).

## Discussion

In this study, we explored the relationship between the gut microbiome and the HIV-1 reservoir in PLWH on long-term ART, identifying microbial patterns and metabolic pathways associated with reservoir size. Interestingly, we observed lower bacterial evenness in individuals with a smaller intact proviral reservoir size in blood. Furthermore, *Lachnospira sp000437735* and *Phocaeicola spp.*, species related to gut health [39, 40], were more abundant in individuals with a smaller intact reservoir and smaller intact-to-total proviral ratio, respectively, and negatively correlated with reservoir size and intact-to-total proviral ratio. Conversely, *Prevotella spp.*, species described in chronic inflammation [9, 41–43], were positively correlated with intact reservoir size, and were also enriched in the group with a high ratio. The microbial metabolic pathways of glycolysis and branched-chain amino acid biosynthesis were significantly enriched in individuals with a larger intact reservoir.

Following the initiation of ART, the intact proviral reservoir declines slowly over time, accompanied by an accumulation of defective proviruses, resulting in a progressively lower intact-to-total proviral ratio. The most pronounced decay of the intact reservoir typically occurs within the first four to seven years after ART initiation, after which the decline reaches a plateau [44–47]. In our cohort, individuals with a smaller intact reservoir size had a significantly higher current and nadir CD4^+^ T cell count and CD4^+^/CD8^+^ ratio compared to those with a larger intact reservoir, however, the duration of ART did not differ between the groups. Our cohort consisted of a diverse group of PLWH with an almost equal distribution of sexes and sexual orientation, which strengthens our results.

We observed lower alpha diversity, as measured by Shannon and Simpson indices, but not in species richness (Observed), in individuals within the group with a smaller intact proviral reservoir. This indicates a difference in evenness. Yet, regression analysis did not reveal a statistically significant linear association between individual intact reservoir size and alpha diversity. This observed trend aligns with findings from a previous study, which also reported reduced diversity in individuals with viral control and presumed smaller reservoir size [38]. On the other hand, studies have shown that individuals with higher CD4^+^ T cell counts, and elite controllers have a higher microbial diversity [18, 20]. HIV infection is associated with reduced alpha diversity, but confounding factors of diet, ART, sex, and sexual orientation have notable effects, with a more reduced diversity in females and men who have sex with women (MSW), when compared to men who have sex with men (MSM) and people without HIV [1, 10, 19, 48]. Nevertheless, the associations between microbial diversity and the reservoir remain poorly defined, and the potential mechanisms are not yet understood [18, 21].

Moreover, we identified several bacterial taxa associated with a lower intact HIV-1 reservoir size and a reduced intact-to-total proviral ratio. Notably, we observed a significant enrichment of *Phocaeicola plebeius* in individuals with a smaller intact HIV-1 reservoir and a lower intact-to-total proviral ratio. Additionally, *Phocaeicola massiliensis* and *Phocaeicola plebeius* were both negatively correlated with the intact-to-total proviral ratio. The role of *Phocaeicola* in HIV pathogenesis and reservoir dynamics remains largely unexplored. A previous study [49] reported positive associations between *Phocaeicola* abundance and HIV-1 reservoir size, as well as inflammatory protein levels, though these findings were limited to treatment-naïve individuals. Conversely, *Phocaeicola plebeius* has been shown to decline in abundance with increasing HIV disease severity [50], highlighting potential context-dependent effects. Importantly, *Phocaeicola* species are known for their anti-inflammatory function and roles in gut health with dietary fiber fermentation and polysaccharide metabolism [40, 51].

Furthermore, we found that *Eubacterium G sp000434315* was more abundant in individuals with a smaller intact reservoir and ratio of intact provirus. This observation is consistent with previous studies showing enrichment of *Eubacterium* species in elite controllers [52]. Similarly, *Faecalibacterium prausnitzii* — a prominent butyrate-producing bacterium [53] frequently depleted in PLWH — demonstrated a negative correlation with intact reservoir size in our cohort. Taken together, these findings support the hypothesis that butyrate-producing bacteria, such as *Eubacterium* spp [54]. and *F. prausnitzii*, may play a protective role in modulating HIV reservoir dynamics, potentially by reducing systemic immune activation 55 and promoting mucosal integrity and immune homeostasis.

Additionally, we found that *Lachnospira sp000437735* was enriched and negatively correlated with both intact HIV-1 reservoir size and the intact-to-total proviral ratio. In contrast, *Lachnospira rogosae* was more abundant in individuals with a larger intact reservoir and positively correlated with the intact proviral ratio. While the functional roles of these species remain poorly characterized, their opposing associations suggest distinct influences on reservoir dynamics within the same genus. The *Lachnospiraceae* family is known for fermenting complex carbohydrates and producing short-chain fatty acids (SCFAs), particularly butyrate, which supports gut barrier integrity and immune regulation. However, *Lachnospiraceae* is functionally diverse — some taxa have been linked to inflammation and immune dysregulation — and its overall abundance is often reduced in PLWH, reflecting HIV-associated gut dysbiosis [56, 57].

Moreover, *Prevotella* has been frequently discussed in HIV infection, shown to be associated with sexual orientation, enriched in MSM [10, 48]. However, an association with chronic inflammation, microbial translocation, and reduced CD4^+^ T cell counts has also been observed [8–10, 41–43]. In our cohort, with a high proportion of females and relatively low proportion of MSM, *Prevotella copri* was positively correlated with larger intact HIV-1 reservoir, while *Prevotella bivia* was associated with a higher intact-to-total proviral ratio. These results emphasize the concept that certain *Prevotella* species may contribute to reservoir persistence by promoting immune activation and compromising gut barrier integrity.

In addition to microbial taxa, we also identified metabolic pathways such as glycolysis IV, the super-pathway of branched-chain amino acid biosynthesis (BCAA biosynthesis), and L-valine biosynthesis, which were significantly enriched in individuals with a high intact proviral reservoir size. In host cells, previous studies suggest that glycolysis plays a dual role in HIV-1 infection. Kang et al [58] reported that increased glycolysis in CD4^+^ T cells facilitates viral invasion, latency formation, and inflammation. Conversely, Shytaj et al. [59] demonstrated that glycolysis upregulation supports initial HIV-1 replication in activated cells, but the virus downregulates glycolysis during the transition to latency, enabling it to evade immune responses and antiretroviral therapy. Furthermore, L-valine is one of the three products of BCAA biosynthesis from pyruvate, the end product of glycolysis IV, along with L-leucine and L-isoleucine. Bacterial BCAA biosynthesis depends on the availability of pyruvate, so increased glycolysis can fuel BCAA production. Moreover, BCAA accessibility plays a pivotal role in the activation and function of T cells, influencing the mechanistic target of rapamycin (mTOR) signaling pathway in host cells. Activation of mTOR, in host cells, by BCAAs affects glycolysis, enhances T cell activation and cytokine production, and modulates immune responses [60]. HIV relies heavily on the mTOR pathway, which regulates key signaling and metabolic processes critical for viral entry, replication, and the establishment of latent reservoirs. However, clinical trials targeting mTOR inhibition have not demonstrated a reduction in reservoir size but have shown a decrease in HIV transcription within gut-resident T cells [61–63]. Similarly, CDP-diacylglycerol biosynthesis pathways I and II were enriched in individuals with a high ratio of intact provirus. CDP-diacylglycerol is a key precursor in the biosynthesis of major phospholipids, including phosphatidylglycerol, cardiolipin, and phosphatidylethanolamine [64]. Emerging evidence suggests that alterations in phospholipid metabolism may contribute to a cellular environment conducive to rapid HIV rebound following ART interruption [65] and have also been associated with individuals who fail to control HIV replication.

Our work did not replicate the results reported by Borgognone et al. [38], where the authors described an association between a higher *Bacteroidales*/*Clostridiales* ratio and a smaller total HIV-1 reservoir. In our cohort, we observed a positive trend between both *Bacteroidales* and *Clostridiales* individually — as well as their ratio — and a higher intact-to-total proviral ratio. The differences in reservoir quantification method (total HIV-1 DNA), cohort size, treatment history, and disease course between the two studies may account for the observed discrepancies.

We acknowledge several limitations in our study. First, while we only collected basic dietary information, we did not assess detailed dietary components, such as specific nutrient intake, fasting or non-fasting, or other lifestyle factors (e.g., physical activity, other medications) that could influence gut microbiome composition and, in turn, the HIV reservoir. Second, our analysis of the reservoir was limited to peripheral blood, whereas the majority of the reservoir is thought to reside within lymphoid tissues, including GALT. Third, we did not assess systemic markers of microbial translocation or inflammation in blood collected at the time of fecal and reservoir sampling, which would have strengthened the integration between gut microbiome and metabolic pathway findings and host immune activation. Fourth, although our final sample size was sufficient after applying exclusion criteria, a larger cohort would increase the statistical power and further enhance the generalizability of our results. Finally, the exploratory nature of our cross-sectional study cannot establish directionality or show causality between species, metabolic pathways and the HIV reservoir. Further longitudinal and mechanistic studies are essential to clarify these relationships.

Overall, our findings indicate a link between gut microbiome composition and HIV-1 reservoir size. We show that microbial evenness is lower in individuals with a smaller intact proviral reservoir and observe certain microbial patterns associated with the size and ratio of intact provirus in the blood reservoir. Additionally, we identify key metabolic pathways linked to reservoir size, which provide a potential mechanistic relationship. The association between the microbial markers and HIV reservoir size in PLWH suggests that variations in the microbiome composition in the gut could shape the immune status and vice versa. These findings highlight the complex interplay between microbial communities and viral persistence. Further research is needed to elucidate these relationships, which could offer valuable insights into microbiome-targeted interventions aimed at modulating the HIV reservoir.

## Electronic Supplementary Material

Below is the link to the electronic supplementary material.

Supplementary Material 1

## Data Availability

The datasets generated and analyzed during the current study, including both the gut metadata and the raw shotgun metagenomic sequencing data, are available in the NCBI SRA repository, BioProject ID: PRJNA1263627.
