# Supplementary material for: Linking gut microbiome to HIV-1 reservoir size in people living with HIV
Source: Gut Pathog. 2026 Mar 30;18:29. doi: 10.1186/s13099-026-00828-2 (PMC13063484; doi:10.1186/s13099-026-00828-2)
Supplement: Supplementary file 1 — Supplementary Material 1 [file 13099_2026_828_MOESM1_ESM.docx]

**Supplementary materials**

Supplementary table 1: Characteristics according to ratio of intact to total provirus at inclusion

| **Characteristics** | **High** N = 14*^1^* | **Low** N = 16*^1^* | **Overall** N = 30*^1^* | **p-value***^2^* |
| --- | --- | --- | --- | --- |
| Sex |  |  |  | 0.7 |
| Male | 9 (64%) | 9 (56%) | 18 (60%) |  |
| Female | 5 (36%) | 7 (44%) | 12 (40%) |  |
| Age (years) | 56 (44, 68) | 50 (44, 60) | 54 (44, 68) | 0.3 |
| CD4^+^ T-cell count | 560 (270, 730) | 640 (470, 685) | 630 (290, 720) | 0.8 |
| Nadir CD4^+^ T-cell count | 160 (70, 400) | 310 (179, 431) | 261 (80, 410) | 0.4 |
| CD4^+^/CD8^+^ ratio | 0.87 (0.40, 1.74) | 1.09 (0.69, 1.38) | 1.05 (0.55, 1.40) | 0.6 |
| HIV RNA <50 copies/ml | 12 (86%) | 15 (94%) | 27 (90%) | 0.5 |
| Duration of ART (years) | 9 (4, 15) | 8 (4, 18) | 8 (4, 15) | 0.5 |
| ART regimen |  |  |  | 0.6 |
| INSTI-based | 11 (79%) | 14 (88%) | 25 (83%) |  |
| NNRTI-based | 3 (21%) | 2 (13%) | 5 (17%) |  |
| BMI (kg/m^2^) | 25.1 (21.0, 30.0) | 24.5 (22.9, 26.8) | 25.0 (22.9, 27.0) | 0.7 |
| Diet |  |  |  | 0.7 |
| Omnivorous | 13 (93%) | 15 (94%) | 28 (93%) |  |
| Others/Unknown | 1 (7.1%) | 0 (0%) | 1 (3.3%) |  |
| Vegetarian | 0 (0%) | 1 (6.3%) | 1 (3.3%) |  |
| Ethnicity |  |  |  | 0.4 |
| Asian | 2 (14%) | 4 (25%) | 6 (20%) |  |
| Black | 1 (7.1%) | 3 (19%) | 4 (13%) |  |
| Caucasian | 11 (79%) | 8 (50%) | 19 (63%) |  |
| Latino | 0 (0%) | 1 (6.3%) | 1 (3.3%) |  |
| Route of transmission |  |  |  | 0.3 |
| Heterosexual | 7 (50%) | 11 (69%) | 18 (60%) |  |
| MSM | 7 (50%) | 5 (31%) | 12 (40%) |  |
| Intact provirus | 193 (72, 391) | 36 (31, 53) | 60 (34, 196) | <0.001 |
| 5´defective provirus | 265 (138, 997) | 306 (163, 388) | 281 (142, 832) | 0.9 |
| 3´defective provirus | 282 (96, 902) | 143 (110, 257) | 190 (104, 342) | 0.2 |
| Total defective provirus | 530 (229, 1,899) | 480 (274, 701) | 527 (272, 1,099) | 0.8 |
| Total provirus | 679 (282, 2,230) | 526 (310, 762) | 632 (307, 1,138) | 0.4 |
| Ratio intact-to-total provirus | 0.20 (0.18, 0.29) | 0.09 (0.05, 0.12) | 0.12 (0.08, 0.19) | <0.001 |

| ART, antiretroviral therapy; INSTI, integrase strand transfer inhibitor; NNRTI, non-nucleoside analogue reverse transcriptase inhibitor; MSM, men who have sex with men; BMI, body mass index. CD4^+^ T-cell count (cells/mm^3^); Nadir CD4^+^ T-cell count (cells/mm^3^); provirus (proviral HIV DNA copies/million resting CD4^+^ T-cells). |
| --- |
| *^1^* n (%); Median (Q1, Q3) |
| *^2^* Pearson’s Chi-squared test; Wilcoxon rank sum test; Fisher’s exact test; Wilcoxon rank sum exact test |
| *^3^* False discovery rate correction for multiple testing |

Supplementary Figure 1: Intact Proviral DNA Assay (IPDA) analysis of peripheral blood resting CD4^+^ T cells (rCD4^+^ T cells). (A) Frequencies of intact and defective proviruses per million rCD4^+^ T cells. Total proviruses were estimated with the sum of intact, 5′ defective, and 3′ defective proviruses from each participant. Horizontal lines indicate median values (n=30). Median values as cutoff for low and high groups of intact proviruses (60 copies/10^6^ rCD4^+^ T cells) (B) and ratio of intact to total proviruses (0.13) (C). (D) Correlation of intact to total proviruses per million rCD4^+^ T cells (spearman r=0.68, p<0.0001).

Supplementary Figure 2: Regression analysis plot showing the association between alpha diversity indices and intact proviral reservoir size.

A B

Supplementary Figure 3: Boxplot showing the differences in *Bacteriodales*, *Clostridiales* and *Bacteriodales*/*Clostridiales* ratio between high and low groups based on intact proviral reservoir size (A) and ratio of intact-to-total proviruses (B).
